# Fabrication of Hypericin Imprinted Polymer Nanospheres via Thiol-Yne Click Reaction

**DOI:** 10.3390/polym9100469

**Published:** 2017-09-24

**Authors:** Yuxin Pei, Fengfeng Fan, Xinxin Wang, Weiwei Feng, Yong Hou, Zhichao Pei

**Affiliations:** Shanxi Key Laboratory of Natural Products & Chemical Biology, College of Chemistry & Pharmacy, Northwest A&F University, Yangling 712100, Shaanxi, China; fanfengfeng1314@163.com (F.F.); xinxinwang@nwafu.edu.cn (X.W.); fengweiwei@nwafu.edu.cn (W.F.); houyong2012@126.com (Y.H.)

**Keywords:** molecular imprinting, hypericin, click reaction, nanospheres

## Abstract

To fabricate molecularly imprinted polymer nanospheres via click reaction, five different clickable compounds were synthesized and two types of click reactions (azide-alkyne and thiol-yne) were explored. It was found that molecularly imprinted polymer nanospheres could be successfully synthesized via thiol-yne click reaction using 3,5-diethynyl-pyridine (**1**) as the monomer, tris(3-mercaptopropionate) (tri-thiol, **5**) as the crosslinker, and hypericin as the template (MIP–NSHs). The click polymerization completed in merely 4 h to produce the desired MIP–NSHs, which were characterized by FTIR, SEM, DLS, and BET, respectively. The reaction conditions for adsorption capacity and selectivity towards hypericin were optimized, and the MIP–NSHs synthesized under the optimized conditions showed a high adsorption capacity (*Q* = 6.03 μmol·g^−1^) towards hypericin. The imprinting factors of MIP–NSHs towards hypericin, protohypericin, and emodin were 2.44, 2.88, and 2.10, respectively.

## 1. Introduction

As an important type of artificial receptor, molecularly imprinted polymers (MIPs) [1,2], proposed in the latter part of the last century, have become increasingly attractive to both the academic community and in industry due to their high affinity towards given molecules (templates), high chemical stability, and low cost [3], and have been applied widely in chromatography [4], solid phase extraction (SPE) [5], separation [6], immunoassay [7], sensors [8], catalysis [9], and drug delivery [10,11,12]. MIPs are synthesized via the copolymerization of functional monomers and crosslinkers in the presence of template molecules that form assemblies with functional monomers driven by covalent or non-covalent interactions. Sequential removal of the template molecules from the crosslinked polymer networks leaves the recognition cavities complementary to the shape, size, and position of the functional groups, which then show specific recognition and rebinding capacity towards the template molecules upon re-exposure [13].

The most common physical form of MIPs is polymer monolith obtained via bulk polymerization, which usually produces irregular polymeric particles in a range of 5–100 μm in low yield (less than 50%) [13,14] through a tedious process of crushing, grinding and sieving. Although the method is simple, it causes considerable wastage of the MIPs. More importantly, the irregularities of the resultant particles, in terms of size, shape, and architecture, produced by this method make sample handling difficult and decrease separation efficiency, which limit the application of MIPs, particularly in SPE or chromatography [15,16,17]. In contrast, spherical particles with a well-defined structure and monodispersity are more desirable due to their fast mass transfer rate, better separation performance and low backpressure as solid phase in chromatography column [18,19]. To this end, new synthetic methodologies have been important research topics in the past two decades [13]. Consequently, several methods have been successfully established for the fabrication of spherical MIPs beads, including inverse suspension polymerization [20], emulsion polymerization [21], solution polymerization [22], multi-step swelling polymerization [23], and precipitation polymerization [13,24]. However, these methods are either complicated in manipulation, require a long time to complete polymerization, or require surfactants (which are difficult to remove from MIPs) to prevent aggregation of polymer particles formed in the process of polymerization. Development of new methods that can produce MIP nanospheres efficiently with well-defined architecture in a high yield is therefore still much desired.

Click reaction, introduced by Sharpless in 2001 [25], has been recognized as a popular and powerful tool in controlling macromolecular architecture due to its mild, fast, highly efficient and specific reaction characteristics [26]. However, click reaction was not applied in the field of molecular imprinting technology until 2011, when Ye and his co-workers [27] prepared clickable molecularly imprinted core-shell nanoparticles using a simple one-pot precipitation polymerization with sequential addition of monomers containing clickable functional groups. To this day, few reports of click reactions for the synthesis of MIPs have been published in the literature. Examples of this include Xu et al. who prepared 2D molecularly imprinted materials based on mesoporous silicas via click reaction [28], and Mendes et al. who fabricated glycoprotein selective surfaces by using the click-imprinting technique [29]. Extensive application of click reactions in the fabrication of MIPs might be restricted by the shortage of commercially available clickable monomers and crosslinkers.

**Scheme 1:**
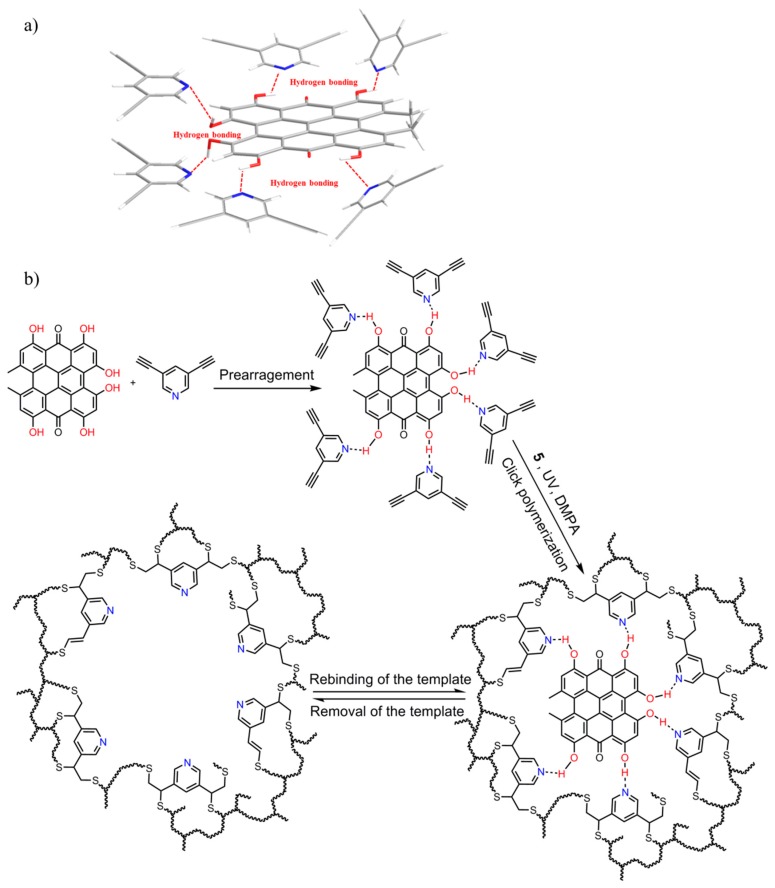
(**a**) Illustration of hydrogen bonding of template, hypericin with functional monomer **1**; (**b**) Schematic illustration of fabricating MIP–NSHs with **1** as monomer and **5** as crosslinker.

Previously, with ultrasonic assistance, our group developed a one-step approach to synthesize polymeric nanospheres via click reaction between azide and alkyne [30,31] in the absence of surfactants. The polymerization was completed in 4 h and the size of the nanospheres were controlled by varying the combination of monomers and crosslinkers and reaction conditions. Meanwhile, hypericin is a photosensitive antiviral, anticancer and antidepressant agent derived from *Hypericum perforatum* (*St John’s wort*) by multistep extraction, therefore, the development and application of hypericin imprinted polymer can speed up and improve the isolation process or can be used in a sensor for hypercin monitoring in biological samples [32]. In this work, we explored the possibility of using click reaction to synthesize molecularly imprinted nanospheres with hypericin as the template. To our delight, molecularly imprinted nanospheres towards hypericin (MIP–NSHs) were successfully obtained by using 3,5-diethynyl-pyridine (**1**) as the functional monomer, trimethylolpropane tris(3-mercaptopropionate) (tri-thiol, **5**) as the crosslinker in the presence of hypericin (see Scheme 1). The thiol-yne click polymerization only took a mere 4 h to yield the desired MIP–NSHs. The MIP–NSHs prepared under the optimized conditions displayed a high adsorption capacity (*Q* = 6.03 μmol·g^−1^) and fair selectivity towards hypericin. This work presents a straightforward method to fabricate molecularly imprinted nanospheres via thiol-yne click reaction.

## 2. Experimental Section

### 2.1. Chemicals and Instrumentation

The chemicals 3,5-dibromopyridine (98%), 2,6-dibromopyridine (98%) were purchased from Daejeon Fung Teng Co., Ltd. (Beijing, China). The chemical 2-methyl-3-butyn-2-ol (98%) was purchased from J & K (Beijing, China). Copper(I) Iodide (98%), trimethylolpropane (>98%), pentaerythritol (98%), bis(triphenylphosphine)palladium(II) chloride (Pd 15.2%), and sodium methylate (97%) were purchased from Aladdin (Shanghai, China). Trimethylsilylacetylene (98%) was purchased from Energy Chemical (Shanghai, China). Benzoin dimethyl ether (DMPA) (98%) was purchased from Heowns (Tianjin, China). Sodium azide (99%) was purchased from Xiya Reagent Co., Ltd. (Chengdu, China). Potassium hydroxide, acetonitrile, acetone, DMSO were purchased from Kelon Chemical Co., Ltd. (Chengdu, China). Toluene, triethylamine, methanol, sulfuric acid (98%), and all the other reagents were purchased from Tianjin Bodi Chemical Industry Co, Ltd. (Tianjin, China). Emodin was purchased from Tianfeng Biological Technology Co, Ltd. (Xi’an, China). Protohypericin (Protohyp) and hypericin (Hyp) were synthesized according to the procedures developed in our lab [33], and characterized by ^1^H NMR (see Supplementary Materials Figures S14 and S15, respectively).

NMR spectra were recorded on a Bruker 500 MHz Spectrometer (Bruker, Fällanden, Switzerland) with working frequencies of 500 MHz for ^1^H in DMSO-*d*_6_, CDCl_3_, or MeOH-*d*_4_. The residual signals from DMSO-*d*_6_ (^1^H: δ 2.50 ppm), CDCl_3_ (^1^H: δ 7.26 ppm), or MeOH-*d*_4_ (^1^H: δ 3.31 ppm) were used as internal standards. Dynamic light scattering (DLS) analysis was performed on a DelsaTM Nano system (Beckman Coulter, Brea, CA, USA). The surface area and the porosity of the prepared polymeric nanospheres were determined using nitrogen physisorption based on the Brunauer-Emmet-Teller (BET) method (ASAP 2020 HD88, Micromeritics Inc., Norcross, GA, USA). Samples were vacuum-degassed at 50 °C for 9 h before the adsorption experiments. Field emission scanning electron microscopy (FESEM) images were obtained with a Hitachi-S4800 instrument (Hitachi, Tokyo, Japan) operating at 10 kV. High performance liquid chromatography (HPLC) was performed with C18 reversed-phase column (5 μm, 4.6 mm × 150 mm, Inertsil ODS-SP, Shimadzu, Tokyo, Japan) at 25 °C. The mobile phase consisted of 50% acetonitrile, 50% of the mixture of ammonium acetate-acetic acid buffer (0.3 M, pH = 6.96), and methanol (1:4, *v*/*v*); detection wavelength: 590 nm; flow rate: 0.4 mL/min; injection volume: 10 μL. The retention time of hypericin under the given chromatographic system is 15.21 min.

### 2.2. Synthesis of Monomers and Crosslinkers

**Figure 1:**
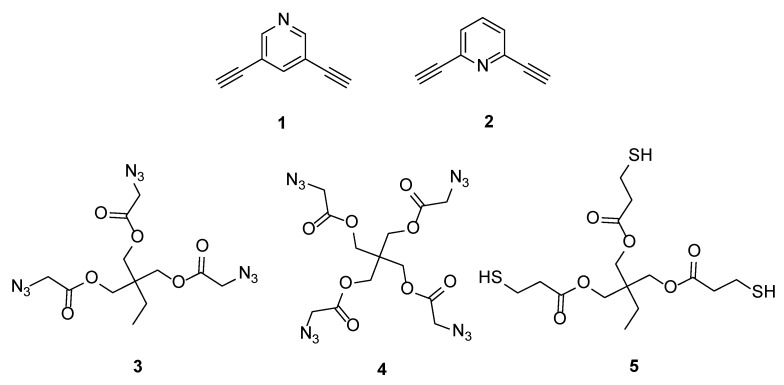
The chemical structures of monomers (**1**–**2**) and crosslinkers (**3**–**5**) used for synthesis of MIP–NSHs.

As phenol hydroxy groups in hypericin are slightly acidic, we chose 3,5-diethynyl-pyridine (**1**) and 2,6-diethynyl-pyridine (**2**) as the monomers. Three crosslinkers of 1,3-bis(azidoacetoxy)-2-azidoacetoxymethyl-2-ethylpropane (tri-azide, **3**), 2,2-bis((2-azidoacetoxy)methyl)propane-1,3-diyl bis(2-azidoacetate) (tetra-azide, **4**), and trimethylolpropane tris(3-mercaptopropionate) (tri-thiol, **5**) were chosen for azide-alkyne or thiol-yne click reactions in this work (see Figure 1). **1**–**5** were synthesized by following the published procedures [30,34,35,36,37] and characterized by ^1^H NMR. The synthetic details can be found in Supplementary Materials and their ^1^H NMR spectra were shown in Figure S1–S5, S9–S13).

### 2.3. Preparation of Imprinted Polymer Nanospheres towards Hypericin and Non-Imprinted Polymer Nanospheres 

Typically, polymer nanospheres imprinted towards hypericin (MIP–NSHs) were prepared as follows. To a quartz tube, 0.1 mmol functional monomer, 0.1 mmol cross-linker, 0.031 mmol DMPA, 2 mL acetone were added, and the mixture was subjected to ultrasonication for 10 min, followed by adding 5 μmol template. The click polymerization was performed for 4 h under UV light at a wavelength of 350 nm at ambient temperature under N_2_ and stirring. After the reaction, the resulting beads were collected, filtered, and extracted to remove hypericin. Then the polymer beads were allowed to dry naturally and grounded with a pestle for 15 min to obtain MIP–NSHs.

The extraction procedure: The nanospheres were extracted with a soxhlet extractor using 10% acetic acid in acetone for 48 h, thereafter the nanoshperes were ultrasonicated in 5 mL 20% acetic acid in acetone for 20 min and then centrifuged; the resulting supernatant was monitiored using HPLC. The ultrasonication step was repeated until no hypericin could be detected in the supernatant. 

Control polymer nanospheres (NIP–NSs) were prepared under identical conditions but in the absence of hypericin. 

### 2.4. Determination of Static Adsorption Capacity

To determine the static adsorption capacity, 5 mg of the nanospheres (MIP–NSHs or NIP–NSs) was placed into a 10 mL plastic centrifuge tube and mixed with 5 mL of the test molecule acetone solution (10.0 μM) to allow adsorption at 25 °C for 24 h. The concentration of the test molecule in the supernatant was measured with HPLC using a calibration curve (see Supplementary Materials Figure S6). The adsorption capacity (*Q*, μmol·g^−1^) was calculated according to Equation 1:

(1) $$Q\;=\frac{{(C}_{0}\;-{\;C}_{e\;})\;V}{W}$$

where *C*_0_ and *C*_e_ represent the initial and equilibrium concentrations of the test molecule in acetone (μM), respectively, *V* (L) is the volume of the solution, and *W* (g) is the dry weight of the nanospheres.

The neat adsorption capacity of MIP–NSHs over that of NIP–NSs, defined as the specific adsorption capacity of MIP–NSHs (*Q*_s_) towards hypericin, is calculated according to Equation 2:

*Q*_s_ = *Q*_1_ − *Q*_2_ (2)

where *Q*_1_ and *Q*_2_ are the static adsorption capacity of MIP–NSHs and NIP–NSs (μmol·g^−1^) towards hypericin, respectively.

### 2.5. Kinetic of Template Adsorption

5.0 mg of MIP–NSHs (or NIP–NSs) were mixed with a hypericin acetone solution (12.5 μM, 5 mL) in a 10-mL centrifuge tube. The tube was sealed and shaken in the dark at 25 °C for different time intervals (0.5, 1, 2, 4, 8, and 12 h, respectively). The concentrations of hypericin in the supernatant were determined by HPLC. The respective adsorptions were then calculated according to Equation 1.

### 2.6. Isotherm Adsorption

Six portions of 5.0 mg polymers were weighed into plastic centrifuge tubes and mixed with 5 mL of hypericin acetone solution with different concentrations (2.5, 5.0, 10.0, 12.5, 25, and 50.0 μM), respectively. The mixtures were shaken for 8 h at 25 °C, and the concentrations of hypericin in the supernatants were determined by HPLC. The respective adsorptions were then calculated according to Equation 1. The isotherm adsorption curve was plotted based on the concentrations of hypericin in supernatants versus the amount of hypericin bound to MIP–NSHs.

### 2.7. Selectivity of MIP–NSHs and NIP–NSs for Hypericin

The binding selectivity of MIP–NSHs and NIP–NSs was evaluated by following the method reported [38]. Briefly, 5 mg of the MIP–NSHs or NIP–NSs were incubated respectively with 5 mL of hypericin, protohypericin, and emodin solution (12.5 μM in acetone) at 25 °C. After incubation under continuous shaking for 24 h, the amounts of hypericin, protohypericin, and emodin bound to the MIP–NSHs or NIP–NSs were measured, respectively. The binding selectivity of the NSs towards different molecules was compared using the “selectivity factor” (*SF*) and “imprinting factor” (*IF*) [39], which are determined as the ratio of distributions and can be calculated by the following equations:

(3) $$D=\frac{C_{0}{\;-\;C}_{e}}{C_{e}}$$

(4) SF=DMIPhDMIP′=(C0, MIPh − Ce, MIPh)Ce, MIPh(C0, MIP′ − Ce, MIP′)Ce, MIP′

where *D* is distribution (L·g^−1^), $D_{\mathrm{MIP}}^{h}$ is distribution of hypericin for MIP (L·g^−1^), $D_{\mathrm{MIP}}^{\prime }$ is distribution of competitor for MIP (L·g^−1^), $C_{0,\;\mathrm{MIP}}^{h}$ is the initial concentration of hypericin for MIP (μM), $C_{e,\;\mathrm{MIP}}^{h}$ is the equilibrium concentration of hypericin for MIP (μM), $C_{0,\;\mathrm{MIP}}^{\prime }$ is the initial concentration of competitor for MIP (μM), and $C_{e,\;\mathrm{MIP}}^{\prime }$ is the equilibrium concentration of competitor for MIP (μM).

(5) IF= DMIPDNIP = (C0, MIP − Ce, MIP)Ce, MIP(C0, NIP − Ce, NIP)Ce, NIP

where $D_{\mathrm{MIP}}$ is the distribution of the test molecule for MIP (L·g^−^^1^), $D_{\mathrm{NIP}}$ is the distribution of the test molecule for NIP (L·g^−^^1^), $C_{0,\;\mathrm{MIP}}$ is the initial concentration of the test molecule for MIP (μM), $C_{e,\;\mathrm{MIP}}$ is the equilibrium concentration of the test molecule for MIP (μM), $C_{0,\;\mathrm{NIP}}$ is the initial concentration of the test molecule for NIP (μM), and $C_{e,\;\mathrm{NIP}}$ is the initial concentration of the test molecule for NIP (μM).

### 2.8. The Reusability of MIP–NSHs

The reusability of MIP–NSHs was evaluated by an adsorption–extraction cycle experiment. One adsorption–extraction cycle consisted of loading the template and reaching equilibrium adsorption, followed by the extraction of the template. For the adsorption, 5 mg of the MIP–NSHs were incubated with 5 mL of hypericin solution (12.5 μM in acetone) at 25 °C. After incubation under continuous shaking for 8 h, the concentration of hypericin in the supernatant was measured by HPLC. The adsorption capacity was calculated according to Equation 1. The MIP–NSHs were subjected to the extraction procedure mentioned above, and then used for the next cycle. 

## 3. Results and Discussion

### 3.1. Synthesis of MIP–NSHs 

#### 3.1.1. Screening of Monomers and Crosslinkers

Previously, we have successfully prepared polymeric nanospheres via the alkyne−azide cycloaddition between either of the monomers and either of the cross-linkers, i.e., four “monomer + crosslinker” combinations (**1** + **3** or **4** and **2** + **3** or **4**) initiated by Cu(PPh_3_)_3_Br [30]. However, due to the low polymer yield from the initiation system, a new photoinitiatior of DMPA was used for polymerization in this work. The four combinations were used for the preparation of imprinted polymers (denoted as P1, P2, P3, and P4 correspondingly) towards hypericin. The adsorption study showed that the MIP–NSHs obtained from **1** + **3** (Table 1, P1) gave the highest specific adsorption capacity (*Q*_s_ = 1.91 μmol·g^−1^) among the four polymers, as shown in Table 1. Under the same conditions, *Q*_s_ from **2** + **3** (Table 1, P2) was far lower at 0.33 μmol·g^−1^. The reason may be that the steric hindrance of **2** is greater than that of **1** when forming the hydrogen bond between the N atom in pyridine ring and the phenolic hydroxyl groups in hypericin, which consequently affects the creation of the imprinting sites and thus impacts the recognition towards hypericin. 

In addition, using tetra-azide **4** instead of tri-azide **3** as the crosslinker did not benefit the adsorption of the resulting MIP–NSHs. For example, the *Q*_s_ of P3 synthesized from **1** + **4** was much lower than that of P1. This might be because the polymer formed with **4** as crosslinker was denser than that with **3** as crosslinker, which make the removal and re-binding of hypericin from the polymer more difficult, and, as a result, lowered the adsorption of MIP–NSHs drastically. Thus, a tri-thiol crosslinker **5** was synthesized and combined with **1** to fabricate MIP–NSHs via thiol-yne click reaction (Table 1, P5). As expected, the *Q*_s_ obtained was the best among the five tested combinations, which may be due to the absence of triazole rings and less rigid structure of P5. Therefore, the subsequent studies were based on the MIP–NSHs obtained from combination of monomer **1** and crosslinker **5**.

#### 3.1.2. Characterization of MIP–NSHs

##### FTIR Analysis

**Figure 2:**
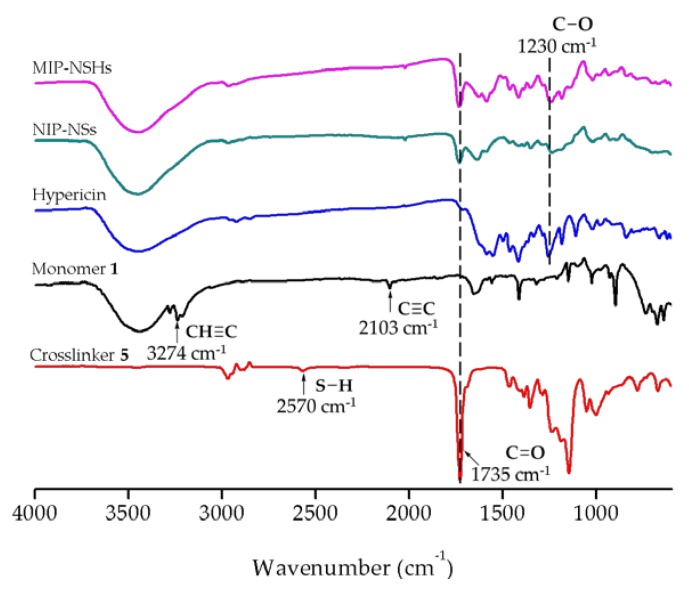
FTIR spectra of MIP–NSHs, NIP–NSs, Monomer **1**, Crosslinker **5** and Hypericin.

To confirm that the thiol-yne click polymerization took place between **1** + **5** in the presence or absence of hypericin, both MIP–NSHs and NIP–NSs were analyzed using FTIR spectroscopy. The IR spectra are shown in Figure 2. In both scenarios, the characteristic absorption of C=O from **5** at 1735 cm^−1^, 2103, 3274, and 2570 cm^−1^ ascribed to the absorptions of C≡C, C–H in alkynyl groups on **1** [30] and S–H on **5**, respectively were not observed on the spectra of the polymers (MIP–NSHs and NIP–NSs). Moreover, the absorption band of C–O on hypericin at 1230 cm^−1^ [38] was found on the spectrum of MIP–NSHs. The results indicated that the click reaction of thiol-yne successfully occurred in both cases.

##### SEM and DLS Analysis

**Figure 3:**
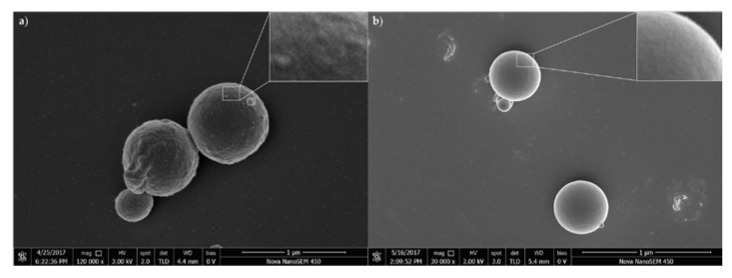
SEM images of the nanospheres from **1** + **5**: (**a**) MIP–NSHs; (**b**) NIP–NSs. The insets are enlarged views correspondingly. Scale bar: 1 μm.

The polymers obtained via click polymerization between **1** + **5** were characterized with FESEM and DLS analyses. The results are shown in Figure 3, Table 2, and Figure S7 (see Supplementary Materials). As shown in Figure 3, both of the imprinted and non-imprinted polymeric nanoparticles showed good spherical morphology. The difference between MIP–NSHs and NIP–NSs was that the surface of the former is much rougher, which can be clearly seen in the enlarged views (insets in Figure 3). DLS analysis showed that the average diameters of the MIP–NSHs and NIP–NSs were 677 and 497 nm, respectively; their polydispersity indexes were 1.137 and 0.994, respectively. In addition, the extraction process yielded an obvious change in the ζ potential of the MIP–NSHs, from 0.95 to −13.51 mV; in contrast, the ζ potential of NIP–NSs was less affected (see Table 2). This is in accordance with what has been reported previously in the literature [38]. The difference in the ζ potentials of MIP–NSHs before and after the extraction process is most likely due to the removal of hypericin molecules from the MIP–NSHs. All these results confirm that the MIP–NSHs can be prepared via thiol-yne click reaction of **1** + **5**.

##### BET Analysis

The average pore diameter, surface area and pore volume of the MIP–NSHs and NIP–NSs fabricated in this work were characterized with BET analysis. The results are summarized in Table 3 and Figure S8 (see Supplementary Materials). As can be seen, the average pore diameter and the pore volume of the MIP–NSHs were 52.839 nm and 0.033 cm^3^·g^−1^, respectively; which are much larger than those of NIP–NSs, indicating that the former has a porous structure, and the latter a compact one. The average pore diameter, surface area and pore volume of the MIP–NSHs were roughly 13, 2.5 and 8.4 times those of the NIP–NSs, respectively. 

#### 3.1.3. Optimization of Preparation Conditions for Specific Adsorption Capacity of MIP–NSHs

In order to obtain MIP–NSHs with the most optimal imprinting effect towards hypericin, the preparation conditions that affected *Q*_s_ were screened and optimized; this included photoinitiator concentration, solvent composition, hypericin concentration, the equivalent ratio of **1** to **5**, etc.

##### Effect of Photoinitiator Concentration on *Q*_s_

As previously mentioned, DMPA was used as the photoinitiator in this work, which generated active free radicals upon exposure to UV light and induced monomer and crosslinker click polymerization. The concentration of DMPA affected not only the rate of polymerization, but also the imprinting effect of the resulting imprinted polymers. The effect of the concentration of DMPA on *Q*_s_ was investigated by varying the concentration of DMPA in a range of 2.0–16 mg·mL^−1^. As shown in Table 4, when the concentration of DMPA increased from 2.0 to 4.0 mg·mL^−1^, *Q*_s_ increased dramatically from 0.65 to 2.71 μmol·g^−1^. However, further increase in the concentration led to the opposite effect with *Q*_s_ decreasing to 0.57 μmol·g^−1^ when the concentration was increased to 16 mg·mL^−1^. Hence, 4 mg·mL^−1^ was used as the optimal concentration of the photoinitiator for subsequent studies.

##### Effect of Solvent Composition on *Q*_s_

Solvent plays an important role in the process of preparing MIP, especially in a non-covalent imprinting process. Proper selection of solvent may promote the formation of non-covalent adducts between functional monomers and templates and enhance the efficiency of imprinting [40]. Considering the solubility of hypericin, acetone was used in this study as a component of the solvent medium for polymerization. The effect of the ratio of acetone to acetonitrile on *Q*_s_ of MIP–NSHs was studied by varying the ratio in a range of 4:0 to 1:3 (*v*/*v*). The results showed that the best ratio of acetone to acetonitrile was 3:1, where *Q*_s_ reached 2.24 μmol·g^−1^ (Table 5); lower ratios led to a dramatic decrease in *Q*_s_, 1.47 μmol·g^−1^ with 1:1, and 0.29 μmol·g^−1^ with 1:3. 

##### Effect of Template Concentration and the Ratio of Monomer to Crosslinker on *Q*_s_

The template concentration and ratio of monomer to crosslinker have a crucial influence on the property of MIPs [41]. They are important factors in dictating the number of binding sites and the rigidity of MIPs [42]. When there is sufficient amount of monomers for the pre-assembly of template and monomer, an increasing amount of template ensures a bigger number of binding sites and therefore a higher adsorption capacity, while an excessive crosslinker concentration (or too low ratio of monomer to crosslinker) leads to a highly crosslinked polymer, which impedes the template to transport in and out of the binding sites and thus decreases the adsorption capacity. This theory was further confirmed in this work. As shown in Table 6, when the concentration of hypericin increased from 0.625 to 2.5 mg·mL^−1^, *Q*_s_ increased from 1.35 to 2.57 μmol·g^−^^1^, however, further increase in the concentration of hypericin to 5.0 mg·mL^−1^ resulted in a clear decrease of *Q*_s_ (see Table 6 P15). As the equivalent ratio of **1** to **5** decreased from 4:1 to 1:2, *Q*_s_ increased at first, from 0.59 to a maximum value of 2.57 μmol·g^−1^, and subsequently decrease dramatically to 0.45 μmol·g^−1^ when the ratio decreased to 1:2. 

Based on the results above, we conclude that the best combination of monomer/crosslinker for fabrication of MIP–NSHs in this work was **1** + **5**. The MIP–NSHs with an optimal *Q*_s_ value (2.57 μmol·g^−1^) can be prepared under the following conditions: the concentratration of hypericin: 5.0 mM; the concentration of monomer **1** and crosslinker **5** is 50 mM each; DMPA: 15.5 mM; solvent: acetone/acetonitrile in 3:1 (*v*/*v*), room temperature, UV light at 350 nm, 4 h. The MIP–NSH prepared under the optimal conditions described above, i.e. P14, were used for subsequent studies.

### 3.2. Kinetic of Template Adsorption

**Figure 4:**
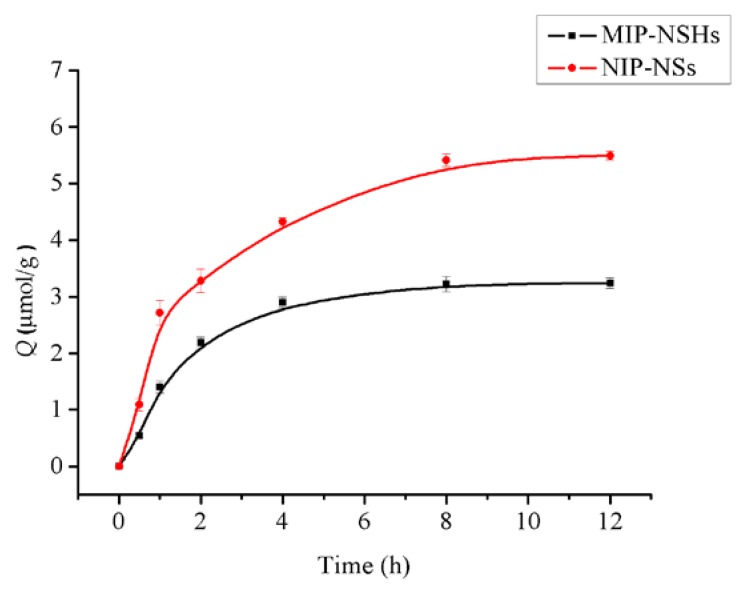
Dynamic adsorption curves of MIP–NSHs and NIP–NSs prepared with the optimal conditions.

The equilibrium adsorption isotherms of MIP–NSHs and NIP–NSs for the binding of hypericin were investigated by batch adsorption experiments. As observed in Figure 4, the adsorption process of the MIP–NSHs displayed two phases: in the first phase, the adsorption amount increased quickly and reached to about one third of the total adsorption capacity during the first hour; and in the second phase, adsorption rate slowed down, and the equilibrium was reached at 8 h. The first phase can be attributed to the binding of hypericin to the recognition sites located on the surface of MIP–NSHs, which bound hypericin molecules at a fast rate. While the second phase can be attributed to the binding of hypericin to the internal binding sites of MIPNSHs, where the diffusion of hypericin from the surface to the inner part of the nanospheres resulted in the slow adsorption rate once the surface recognition sites became saturated. In addition, the equilibrium adsorption capacity of MIP–NSHs is higher than that of NIP–NSs, which indicates a fair imprinting effect. 

### 3.3. Affinity Analysis 

**Figure 5:**
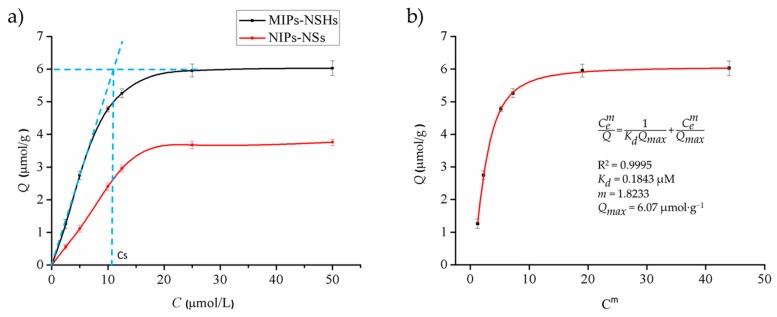
(**a**) Isothermal adsorption curve of MIP–NSHs and NIP–NSs; (**b**) The fitting curve of the extended Langmuir adsorption.

The binding experiments were performed at different initial concentrations of hypericin, ranging from 0 to 50.0 μM, to compare the *Q* of the MIP–NSHs against that of NIP–NSs. Figure 5a shows the binding isotherms for hypericin on the MIP–NSHs and NIP–NSs. It can be seen that the *Q* of MIP–NSHs increased quickly in a linear relationship with the concentration of hypericin before *C*_s_, a critical concentration of 11.5 μM. Thereafter, the increase of the *Q* slowed down and finally reached to a plateau. Furthermore, the amounts of substrate bound to the MIP–NSHs were more than that to the NIP–NSs, which was ascribed to the imprinting effect [43].

The adsorption mechanism study shows that the adsorption of MIP–NSHs towards hypericin fitted well with an extended Langmuir isotherm model (expressed by Equation 5, Figure 5b) [43,44], where *R*^2^ is 0.9995, equilibrium constant *K*_d_ = 0.1843 μM, m is 1.8233, and *Q*_max_ is 6.07 μmol·g^−1^. The results indicate the absorption may be described by monolayer sorption on a non-smooth surface.

(6) $$\frac{C_{e}^{m}}{Q}=\frac{1}{K_{d}\cdot Q_{\max}}\;+\;\frac{C_{e}^{m}}{Q_{\max}}$$

*K*_d_ is the equilibrium constant (μM), *C*_e_ is the equilibrium concentration of hypericin (μM) in supernatant, and *Q*_max_ is the apparent maximum absorption capacity of binding sites (μmol·g^−1^).

### 3.4. Binding Selectivity

**Figure 6:**
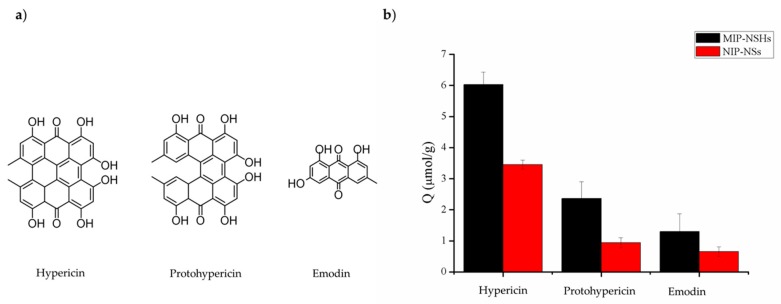
(**a**) The chemical structures of hypericin, protohypericin, and emodin; (**b**) Selective bindings of MIP-NSHs (black) and NIP–NSs (red) toward to hypericin, protohypericin, and emodin, respectively.

The binding selectivity of MIP–NSHs towards hypericin was examined by using a similar method reported previously [38], where protohypericin and emodin were used as competitors of the template (their structures are displayed in Figure 6a). MIP–NSHs or NIP–NSs were incubated respectively with the same amount of hypericin, protohypericin, and emodin under the same conditions. The respective adsorption capacities of MIP–NSHs and NIP–NSs towards the three molecules are shown in Figure 6b. A higher adsorption for hypericin (6.03 μmol·g^−1^) was obtained with MIP–NSHs compared to protohypericin and emodin (2.36 and 1.30 μmol·g^−1^, respectively). The binding selectivity of the NSs was evaluated with *SF* and *IF*, respectively. *SF* of MIP–NSHs towards protohypericin and emodin was 3.34 and 8.04, respectively; *IF* of MIP–NSHs towards hypericin, protohypericin, and emodin, was 2.44, 2.88, and 2.10, respectively (Table 7). 

### 3.5. Reusability of MIP–NSHs

**Figure 7:**
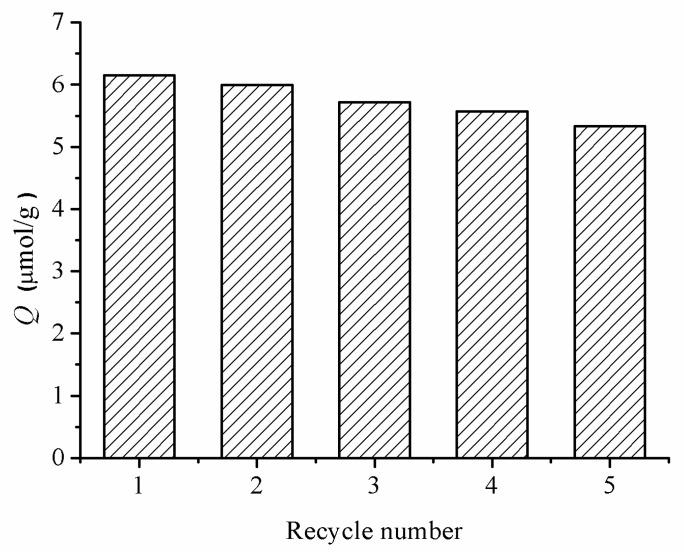
Reusability of MIP–NSHs.

Imprinting materials as chemosensors are robust materials that are supposed to be reused many times, which is essential for reliable, economical and sustainable applications [45]. The evaluation on the reusability of the MIP–NSHs was performed and the results are shown in Figure 7. It can be seen that after five adsorption–extraction cycles, the adsorption capacity of the MIP–NSHs remained high, indicating good stability.

## 4. Conclusions

In conclusion, MIP–NSHs were successfully synthesized via thiol-yne click reaction using 3,5-diethynyl-pyridine as the monomer, tris(3-mercaptopropionate) as the crosslinker, and hypericin as the template. The click polymerization was completed in merely 4 h to produce the desired MIP–NSHs. The reaction conditions for adsorption capacity and selectivity towards hypericin were optimized, and the MIP–NSHs synthesized under the optimized conditions showed a high adsorption capacity (*Q* = 6.03 μmol·g^−1^) and fair selectivity towards hypericin. In addition, MIP–NSHs displayed good reusability up to at least five cycles. This work presents a straightforward method to fabricate molecularly imprinted nanospheres via thiol-yne click reaction. 

## Figures and Tables

**Table 1:** The specific adsorption capacity (***Q_s_***) of the MIP–NSHs towards hypericin.

Polymer	Combination	*Q_s_* (μmol·g^−1^ )	RSD (*n* = 3)
P1	**1** + **3**	1.911	0.022
P2	**2** + **3**	0.331	0.070
P3	**1** + **4**	0.452	0.069
P4	**2** + **4**	0.745	0.038
P5	**1** + **5**	2.204	0.032

**Table 2:** The DLS data of the NSs prepared.

NPs	Paticle Size (nm)	Polydispersity Index	ζ Potential (mV)
MIP–NSHs	677 ± 68	1.137	0.95 ± 0.90
NIP–NSs	497 ± 80	0.994	−2.86 ± 0.52
MIP–NSHs^1^	661 ± 98	0.609	−13.51 ± 0.17
NIP–NSs^1^	464 ± 75	0.874	−1.42 ± 0.56

^1^ The nanospheres after the extracting process.

**Table 3:** The pore size, the surface area, and the pore volume of the NSs.

NPs	Average Pore Diameter (nm)	Surface Area (m^2^·g^−^^1^)	Pore Volume (cm^3^·g^−^^1^)
MIP–NSHs	52.839 ± 0.393	5.274 ± 0.517	0.0330 ± 0.0299
NIP–NPs	4.139 ±0.0522	2.083 ± 0.266	0.00392 ± 0.0726

**Table 4:** The effect of photoinitiator concentration on the specific adsorption capacity.

Polymer	Concentration of DMPA (mg·mL^−1^)	*Q_s_* (μmol·g^−^^1^)	RSD (*n* = 3)
P6	2.0	0.64	0.035
P7	4.0	2.16	0.095
P8	8.0	1.09	0.046
P9	16.0	0.57	0.089

**Table 5:** The effect of solvent composition on *Q_s_*.

Polymer	Acetone/Acetonitrile (*v*/*v*)	*Q_s_* (μmol·g^−1^)	RSD (*n* = 3)
P7	4:0	2.16	0.095
P10	3:1	2.23	0.036
P11	1:1	1.47	0.048
P12	1:3	0.28	0.079

**Table 6:** The effect of template molecule concentration on the specific adsorption capacity.^1^

Polymer	Template concentration (mg·mL^−^^1^)	Ratio of 1 to 5 (in equiv.)	*Q_s_* (μmol·g^−^^1^)	RSD (*n* = 3)
P13	0.625	4:3	1.35	0.059
P7	1.25	4:3	2.17	0.095
P14	2.5	4:3	2.57	0.052
P15	5.0	4:3	1.52	0.039
P16	2.5	4:1	0.59	0.069
P17	2.5	3:1	1.10	0.093
P18	2.5	2:1	2.23	0.058
P19	2.5	1:1	1.94	0.049
P20	2.5	1:2	0.45	0.084

^1^ The solvent used was a mixture of acetone/acetonitrile with a ratio of 3:1 (*v/*v).

**Table 7:** The values of *SF* and *IF*.

Factor	Hypericin	Protohypericin	Emodin
*SF*	/	3.34	8.04
*IF*	2.44	2.88	2.10

## Supplementary Materials

The following are available online at www.mdpi.com/2073-4360/9/10/469/s1, Figure S1: The synthesis of monomer **1**, Figure S2: The synthesis of monomer **2**, Figure S3: The synthesis of crosslinker **3**, Figure S4: The synthesis of crosslinker **4**, Figure S5: The synthesis of crosslinker **5**, Figure S6: (a) Standard curve of hypericin in acetone by HPLC. (b) Standard curve of protohypericin in acetone by HPLC. (c) Standard curve of emodin in acetone by HPLC. HPLC detection conditions: C18 reversed-phase column (5 μm, 4.6 mm × 250 mm, Shimadzu, Japan). The mobile phase consisted of 50% acetonitrile, 50% of the mixture of ammonium acetate-acetic acid buffer (0.3 M, pH = 6.96) and methanol (1:4, *v*/*v*); detection wavelength: 590 nm; flow rate: 0.4 mL/min; injection volume: 10 μL, Figure S7: DLS histograms of MIP–NSHs and NIP–NSs before and after extracting process, Figure S8: The nitrogen adsorption and desorption isotherms of MIP–NSHs and NIP–NSs, Figure S9: The ^1^H NMR spectrum of monomer **1**, Figure S10: The ^1^H NMR spectrum of monomer **2**, Figure S11: The ^1^H NMR spectrum of crosslinker **3**, Figure S12: The ^1^H NMR spectrum of crosslinker **4**, Figure S13: The ^1^H NMR spectrum of crosslinker **5**, Figure S14: The ^1^H NMR spectrum of protohypericin, Figure S15: The ^1^H NMR spectrum of hypericin.
