# Supplementary material for: Fabrication of Hypericin Imprinted Polymer Nanospheres via Thiol-Yne Click Reaction
Source: Polymers (Basel). 2017 Sep 24;9(10):469. doi: 10.3390/polym9100469 (PMC6418589; doi:10.3390/polym9100469)
Supplement: Supplementary file 1 [file polymers-09-00469-s001.pdf]

Supplementary Materials: Fabrication Molecularly Imprinted Polymer Nanospheres *via* Thiol-Yne Click Reaction

Yuxin Pei,* Fengfeng Fan, Xinxin Wang, Weiwei Feng, Yong Hou, and Zhichao Pei*

Shanxi Key Laboratory of Natural Products & Chemical Biology, College of Chemistry & Pharmacy, Northwest A&F University, Yangling, Shaanxi, China;

* Correspondence: peiyx@nwafu.edu.cn; peizc@nwafu.edu.cn; Fax: +86-29-87092769; Tel: +86-29-87091196

1. The Synthesis of monomers and crosslinkers

***3,5-Diethynylpyridine* (1)** [1].

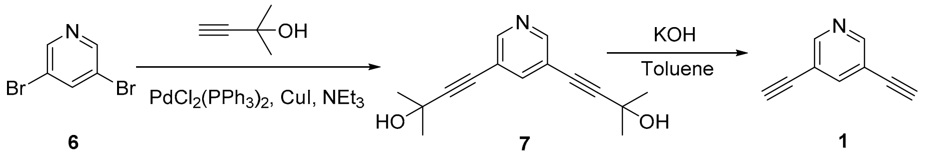

6 7 1

Figure S1. The Synthesis of monomer **1**.

To a 250 mL flask, 3,5-dibromopyridine (98 %) (5.0 g, 21.2 mmol), $PdCl_2(PPh_3)_2$ (450 mg, 0.64 mmol) and CuI (242 mg, 1.27 mmol) were added into 50 mL of Ar-degassed triethylamine and 4.5 mL of Ar-degassed 2-methyl-3-butyn-2-ol (98 %). The resulting solution was stired at room temperature for 24 hrs before the solvent was removed under vacuum. The black residue was extracted with ethyl acetate (15 mL × 5). The organic phase was combined and dried over $MgSO_4$, then filtered through neutral alumina. The filtrate was dried under vacuum to leave a black oil. Purification by column chromatography on silica gel afforded a yellow powder of **7** (4.75 g, 92 %).

To a 250 mL flask, Compound **7** (1.0 g, 4.14 mmol) and KOH (0.46 g, 8.25 mmol) were added into 50 mL of Ar-degassed toluene, the resulting solution was refluxed for 12 hrs. The solvent was removed under vacuum to leave a yellow brown solid, which was then dissolved in dichloromethane. The solution was filtered and the filtrate was dried under vacuum to give a yellow brown solid. Purification by column chromatography on silica gel afforded a white powder of **1** (0.258 g, 49 %). ^{1}H NMR (500 MHz, $CDCl_3$) δ 8.65 (d, *J* = 1.9 Hz, 2H), 7.88 (t, *J* = 1.95 Hz, 1H), 3.26 (s, 2H) ppm.

***2,6-Diethynylpyridine* (2)** [2].

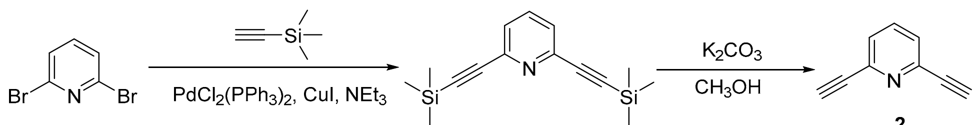

8 9 2

Figure S2. The Synthesis of monomer **2**.

To a 250 mL flask, 2,6-dibromopyridine (98 %) (2.36 g, 10.0 mmol), $PdCl_2(PPh_3)_2$ (351 mg, 0.5 mmol) and CuI (95.5 mg, 0.5 mmol) were added into 100 mL of Ar-degassed triethylamine and 3.2 mL of Ar-degassed trimethylsilylacetylene (22 mmol 98 %), the resulting solution was refluxed for 8 h before the solvent was removed under vacuum. The black residue was extracted with ethyl acetate (30 mL × 5). The organic phase was combined and dried over

$MgSO_4$, then filtered through neutral alumina. The ether was rotary evaporated to leave a black oil, which was purified by column chromatography on silica gel. **9** was otained as a light yellow solid (2.14 g, 79 %).

To a 50 mL flask, Compound **9** (1.02 g, 3.76 mmol) was dissolved in 20 mL of MeOH, followed by addition of K_2CO_3 (2.59 g, 18.8 mmol). The mixture was stirred for 12 h at room temperature. The solvent was evaporated under vacuum. 50 mL CH_2Cl_2 was added to the residue, and the insoluble solids were filtered away. The filtrate was washed with saturated brine (2 × 30 mL), and the organic phase was dried over Na_2SO_4. After removing the solvent, the crude was purified by flash chromatography on silica gel to afford **2** as a white solid (0.358 g, 75 %). ^1H NMR (500 MHz, $CDCl_3$) δ 7.67 (t, J = 7.7 Hz, 1H), 7.46 (d, J = 7.8 Hz, 2H), 3.18 (s, 2H) ppm.

1,3-Bis(azidoacetoxy)-2-azidoacetoxymethyl-2-ethylpropane **(3)** [3].

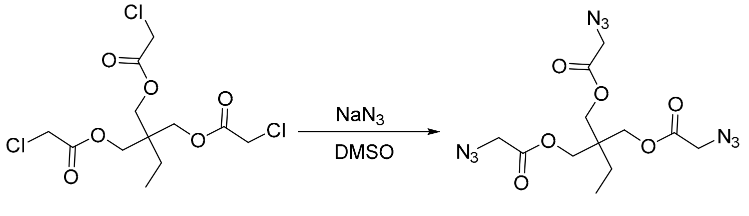

10 **3**

Figure S3. The Synthesis of crosslinker **3.**

To a 25 mL flask, 1,3-Bis(chloroacetoxy)-2-chloroacetoxymethyl-2-ethylpropane (**10,** 1.815 g, 5 mmol) was dissolved in 15 mL DMSO, NaN_3 (1.465 g, 22.5 mmol) was added with cooling. The mixture was heated to 40 °C and stirred for 2 days. After reaction, the mixture system was poured into ice-water, and extracted with CH_2Cl_2 (3 × 15 mL). The CH_2Cl_2 extract was washed 3 times with brine and then dried over Na_2SO_4. Evaporation of the solvent and to leave a yellow liquid, which was purified by column chromatography on silica gel. **3** was otained as a colorless liquid (1.15 g, 60 %). ^1H NMR (500 MHz, $CDCl_3$) δ 4.19 (s, 6H), 3.91 (s, 6H), 1.54 (q, J = 7.6 Hz, 2H), 0.94 (t, J = 7.6 Hz, 3H) ppm.

2,2-Bis((2-azidoacetoxy)methyl)propane-1,3-diyl bis(2-azidoacetate) **(4)** [4]

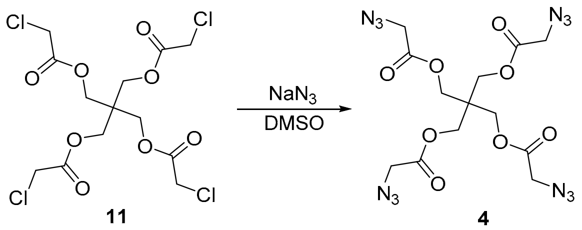

11 **4**

Figure S4. The Synthesis of crosslinker **4.**

To a 50 mL flask, 2,2-Bis((2-azidoacetoxy)methyl)propane-1,3-diyl bis(2-azidoacetate) (2.21 g, 5 mmol) was dissolved in 15 mL DMSO, NaN_3 (1.95 g, 30 mmol) was added with cooling. The mixture was heated to 50 °C and stirred for 2 days. After reaction, the mixture system was poured into ice-water, and extracted with CH_2Cl_2 (3 × 15 mL). The CH_2Cl_2 extract was washed 3 times with brine and then dried over Na_2SO_4. Evaporation of the solvent and to leave a yellow liquid, which was purified by column chromatography on silica gel. **4** was otained as a colorless liquid (1.19 g, 51 %). ^1H NMR (500 MHz, $CDCl_3$) δ 4.30 (s, 8H), 3.94 (s, 8H) ppm.

Trimethylolpropane tris(3-mercaptopropionate) **(5)** [5].

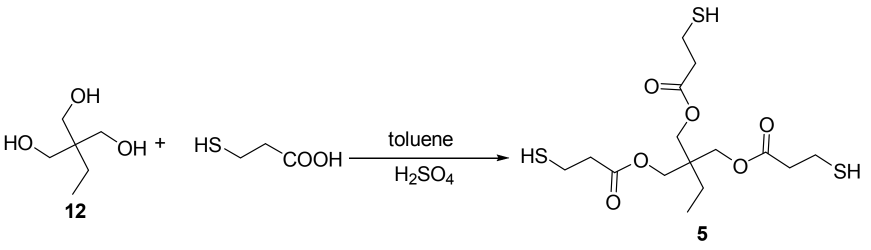

Figure S5. The Synthesis of crosslinker **5.**

1,1,1-tris(hydroxymethyl) propane (3.36 g, 25 mmol), toluene (20 mL), and H_2SO_4 (0.201 g) were added and mixed in a three-neck flask equipped with reflux condensing tube, constant pressure drop funnel, and Dean–Stark trap. 3-mercapto-propanoic acid (8.752 g, 75 mmol) was dropped slowly into the reaction system through the constant pressure drop funnel. The reaction was carried out by heating the ask to 130 °C. Four hours later, the reaction was terminated by cooling down to room temperature. Then, the reaction solution was washed with deionized water to neutrality. The organic phase was separated, and dried over anhydrous $MgSO_4$. Evaporation of the solvent and to leave a colorless and transparent oil (8.957 g, 90 %). ^1H NMR (500 MHz, $CDCl_3$) δ ^1H NMR (500 MHz, $CDCl_3$) δ 4.1 (s, 6H), 2.76 (dd, J = 7.35 and 6.85 Hz, 6H), 2.67 (t, J = 6.65 Hz, 6H), 1.63 (t, J = 8.25 Hz, 3H), 1.51 (q, J = 7.65 Hz, 2H), 0.9 (t, J = 7.55 Hz, 3H) ppm.

2. The standard curves of hypericin, protohypericin and emodin

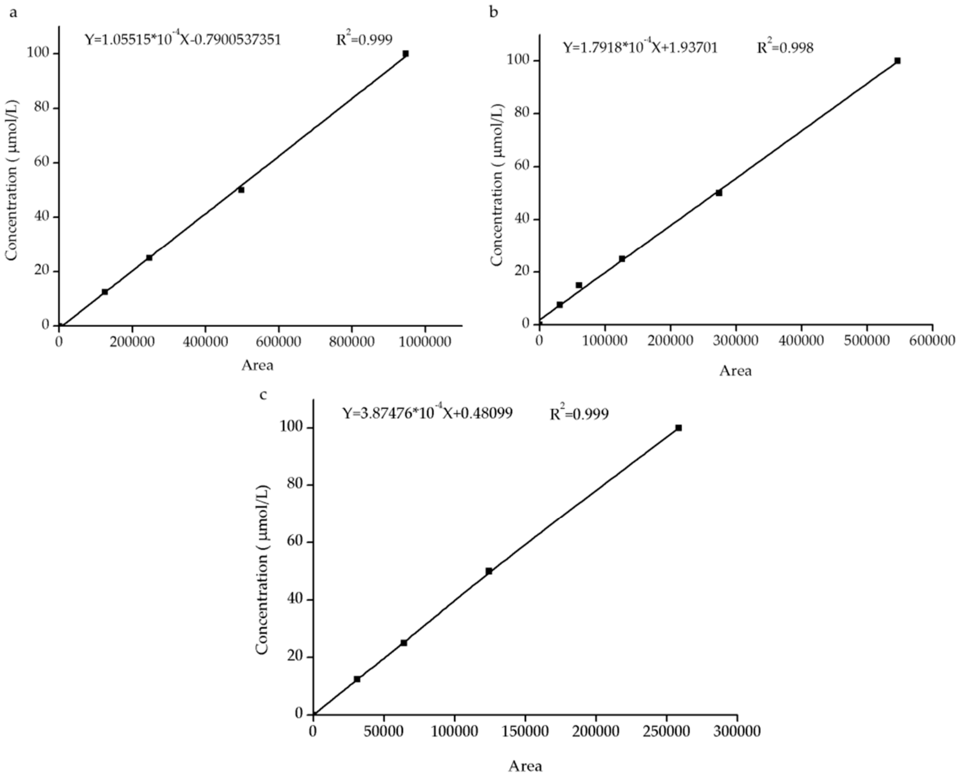

Figure S6. (a) **S**tandard curve of hypericin in acetone by HPLC. (b) Standard curve of protohypericin in acetone by HPLC. (c) Standard curve of emodin in acetone by HPLC. HPLC detection conditions: C18 reversed-phase column (5 μm, 4.6 mm × 250 mm, Shimadzu, Japan).

The mobile phase consisted of 50% acetonitrile, 50 % of the mixture of ammonium acetate-acetic acid buffer (0.3 M, pH = 6.96) and methanol (1:4, v/v); detection wavelength: 590 nm; flow rate: 0.4 mL/min; injection volume: 10 μL.

3. DLS Analysis

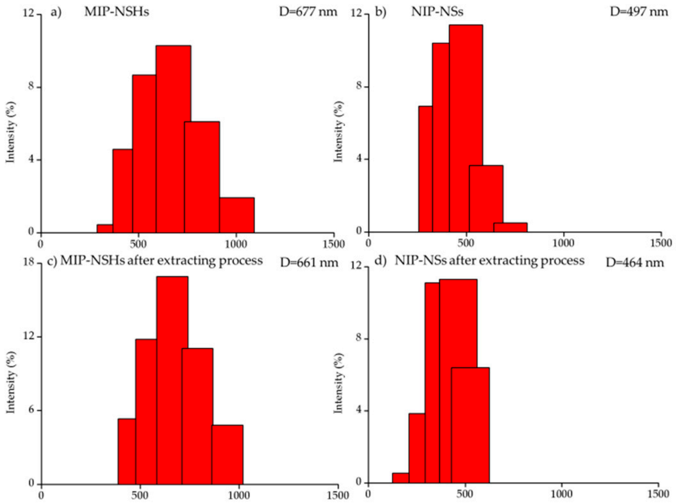

Figure S7. DLS histograms of MIP-NSHs and NIP-NSs before and after extracting process.

4. BET analysis

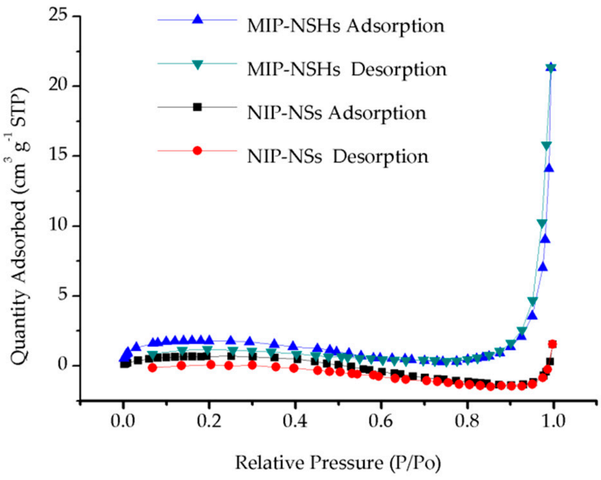

Figure S8. The nitrogen adsorption and desorption isotherms of MIP-NSHs and NIP-NSs.

5. The ^{1}H-NMR spectra of compounds

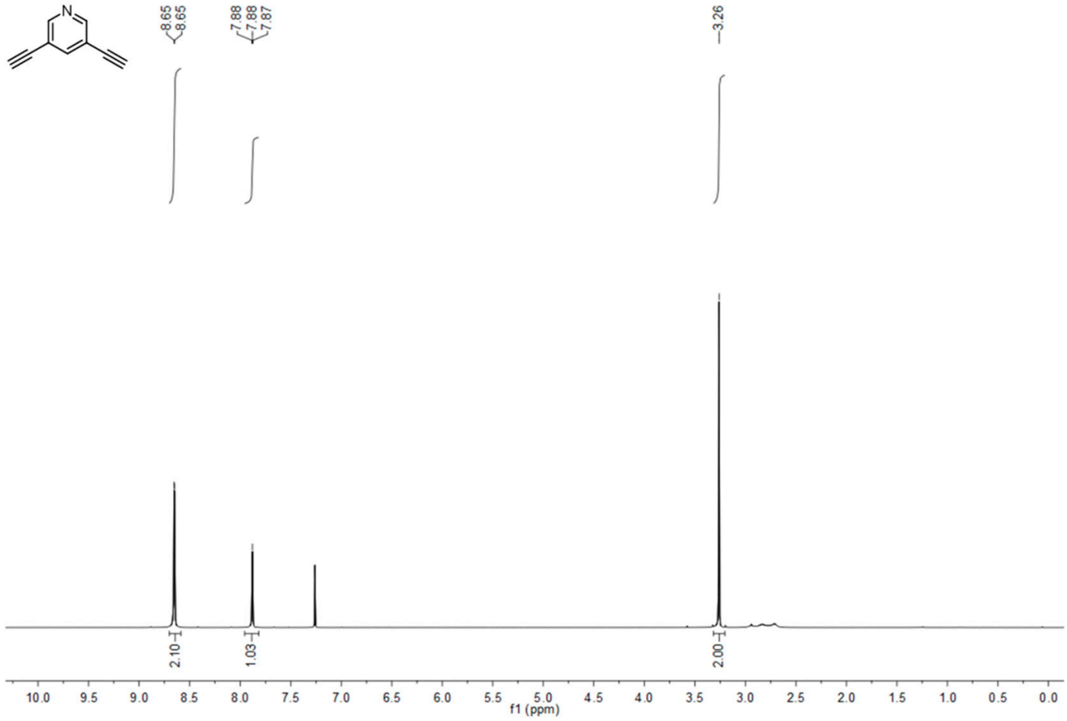

Figure S9. The ^{1}H-NMR spectrum of monomer **1**.

Monomer 1: ^{1}H NMR (500 MHz, $CDCl_3$) δ 8.65 (d, *J* = 1.9 Hz, 2H), 7.88 (t, *J* = 1.95 Hz, 1H), 3.26 (s, 2H) ppm.

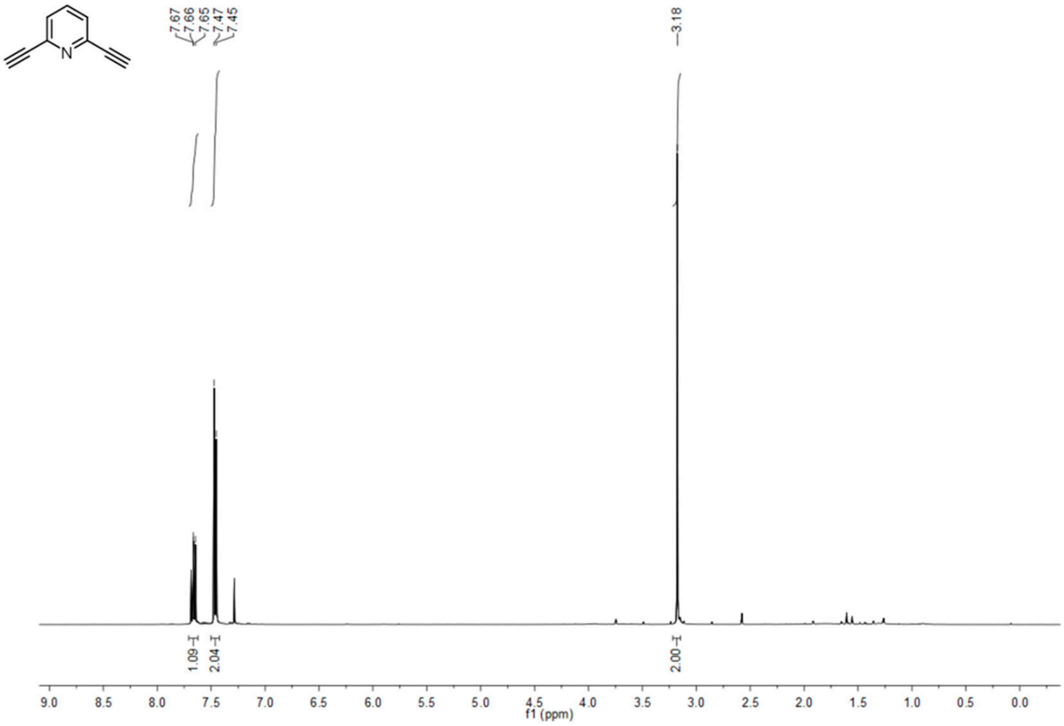

Figure S10. The ^{1}H-NMR spectrum of monomer **2**.

Monomer 2: ^{1}H NMR (500 MHz, $CDCl_3$) δ 7.67 (t, *J* = 7.7 Hz, 1H), 7.46 (d, *J* = 7.8 Hz, 2H), 3.18 (s, 2H) ppm.

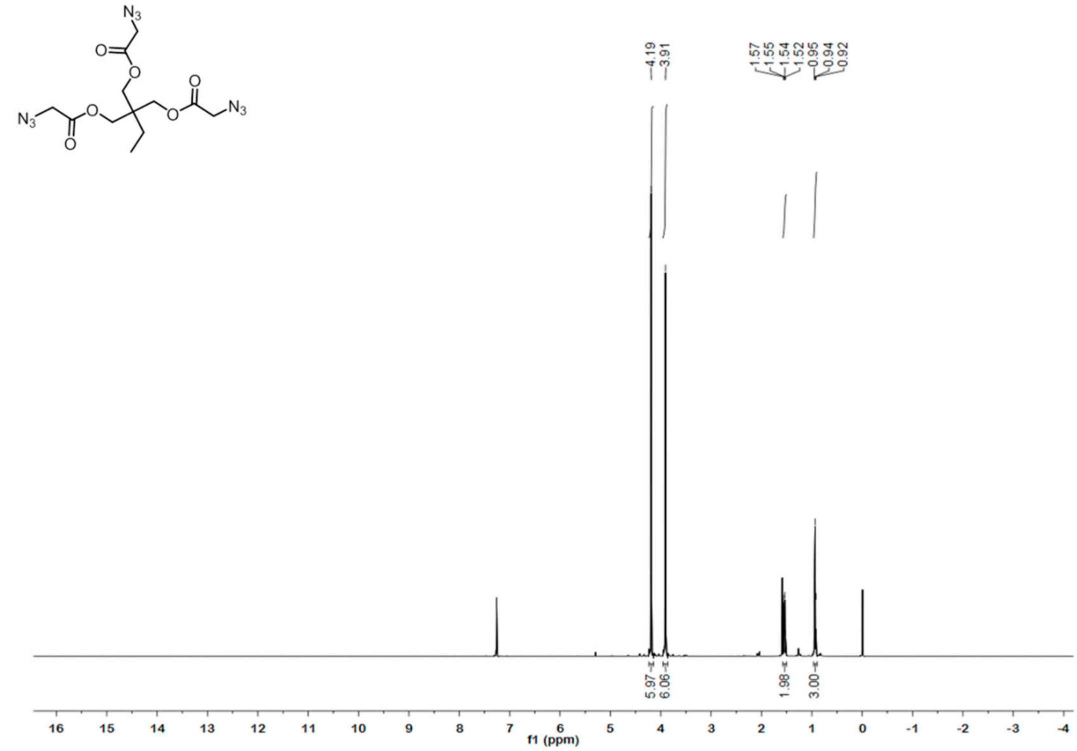

Figure S11. The ¹H-NMR spectrum of crosslinker **3**.

Crosslinker **3:** ¹H NMR (500 MHz, $CDCl_3$) δ 4.19 (s, 6H), 3.91 (s, 6H), 1.54 (q, *J* = 7.6 Hz, 2H), 0.94 (t, *J* = 7.6 Hz, 3H) ppm.

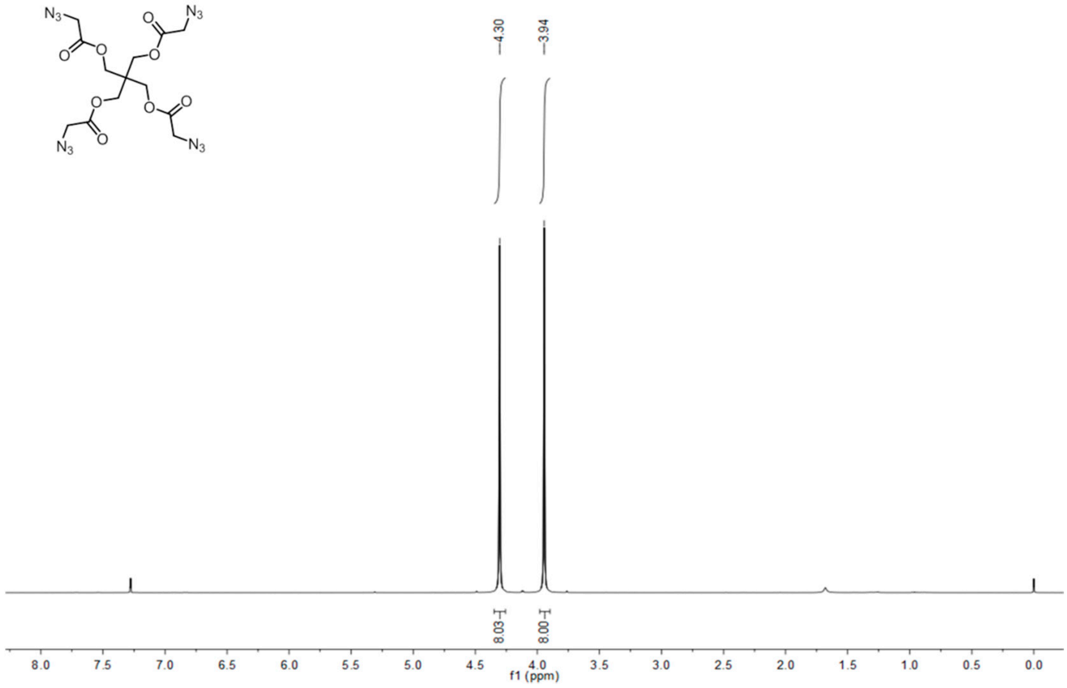

Figure S12. The ¹H-NMR spectrum of crosslinker **4**.

Crosslinker **4:** ¹H NMR (500 MHz, $CDCl_3$) δ 4.30 (s, 8H), 3.94 (s, 8H) ppm.

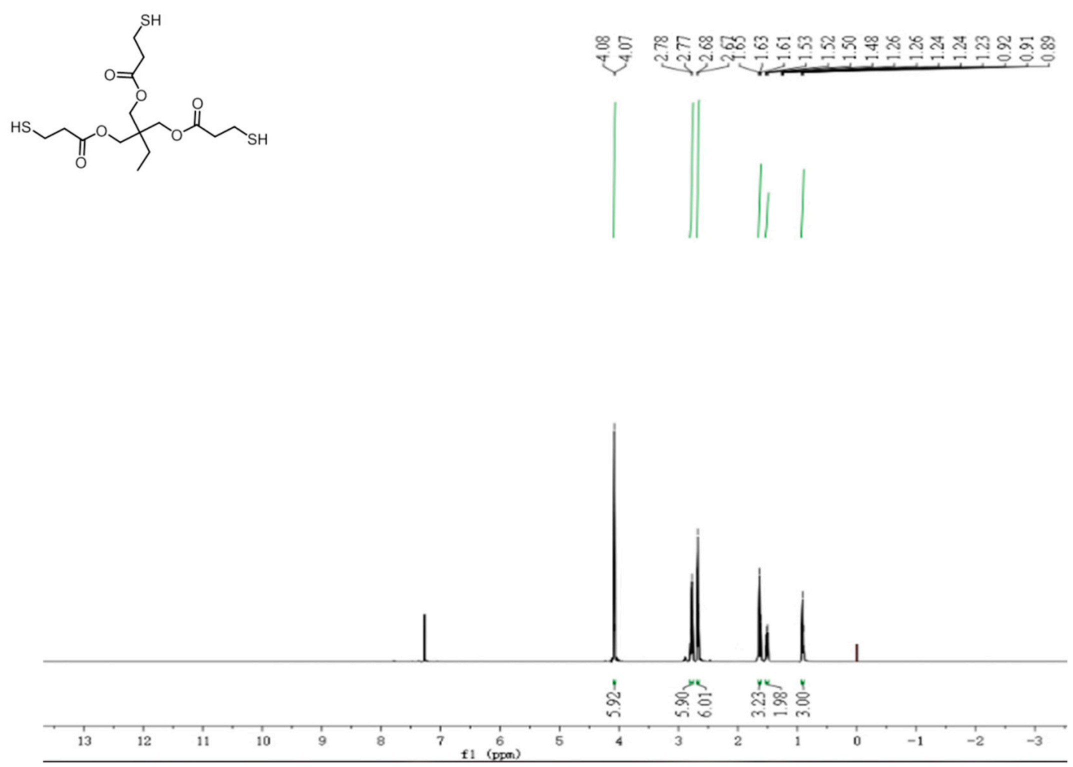

Figure S13. The ^{1}H-NMR spectrum of crosslinker **5**.

Crosslinker **5:** ^{1}H NMR (500 MHz, $CDCl_3$) δ 4.1 (s, 6H), 2.76 (dd, *J* = 7.35 and 6.85 Hz, 6H), 2.67 (t, *J* = 6.65 Hz, 6H), 1.63 (t, *J* = 8.25 Hz, 3H), 1.51 (q, *J* = 7.65 Hz, 2H), 0.9 (t, *J* = 7.55 Hz, 3H) ppm.

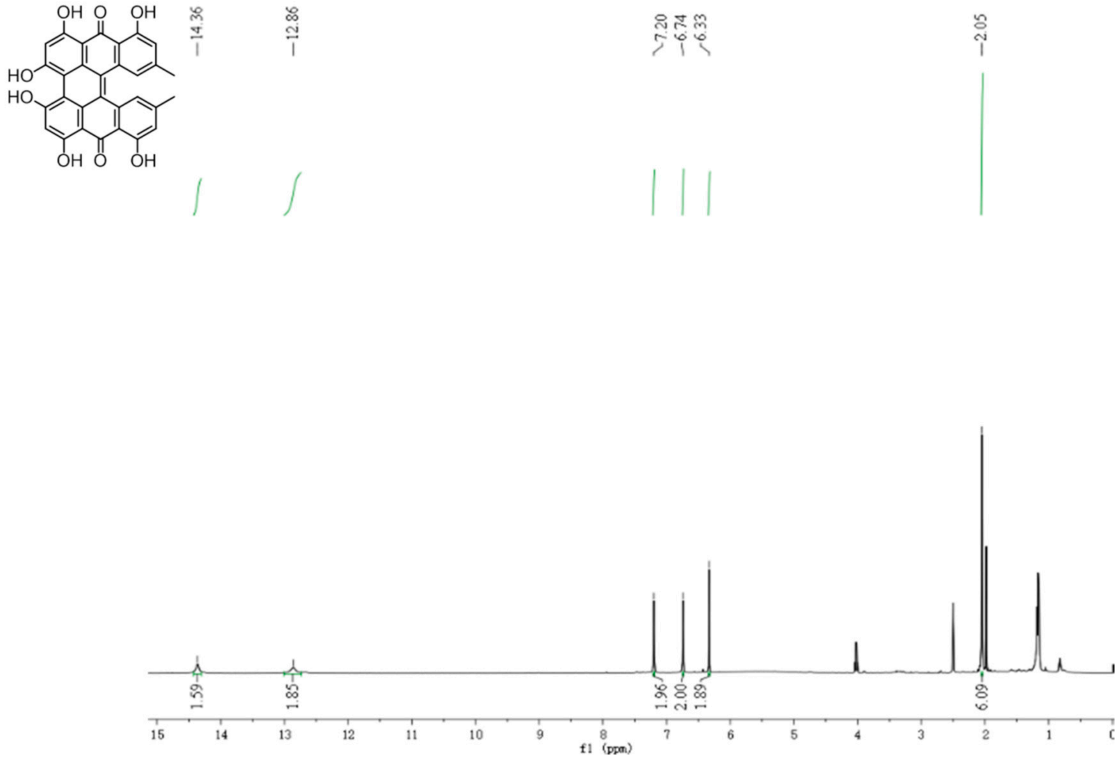

Figure S14. The ^{1}H-NMR spectrum of protohypericin.

Protohypericin: ^{1}H-NMR (500 MHz, DMSO-d_6) δ 14.36 (s, 2H), 12.86 (s, 2H), 7.20 (s, 2H), 6.74 (s, 2H), 6.33 (s, 2H), 2.05 (s, 6H).

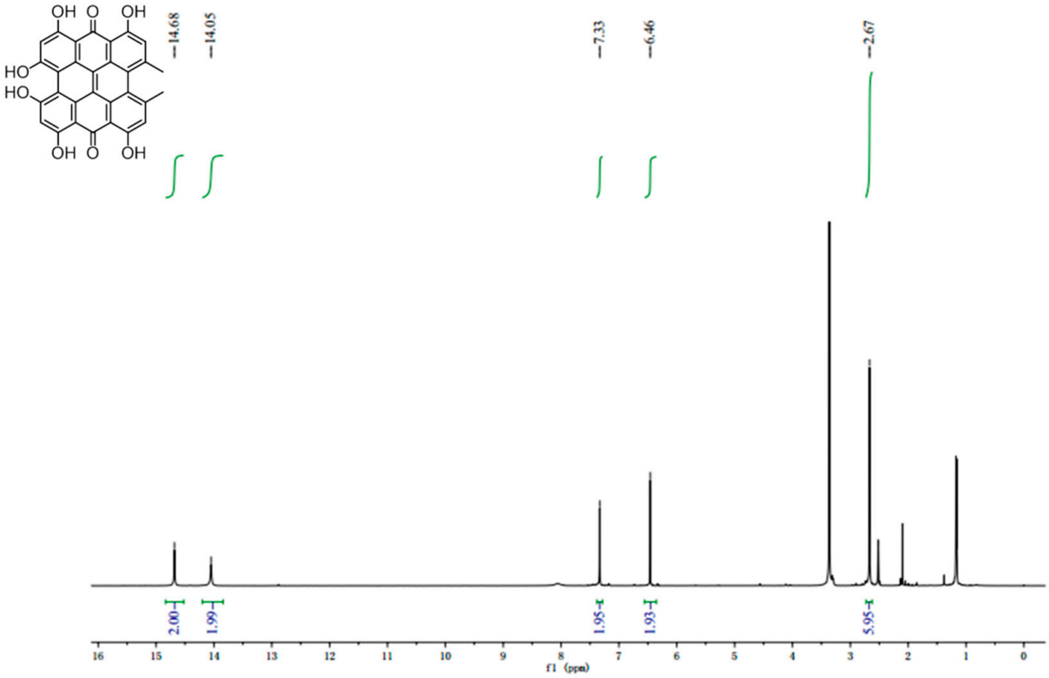

Figure S15. The ^{1}H-NMR spectrum of hypericin.

Hypericin: ^{1}H-NMR (500 MHz, DMSO-d_6) δ 14.68 (s, 2H), 14.0 (s, 2H), 7.33 (s, 2H), 6.46 (s, 2H), 2.67 (s, 6H).

References

1. Sun S S, Lees A J. Synthesis and photophysical properties of dinuclear organometallic rhenium (I) diimine complexes linked by pyridine-containing macrocyclic phenylacetylene ligands. *Organometallics*. **2001**, 20(11), 2353-2358.
2. Dana, B.H.; Robinson, B.H.; Simpson, J. Intramolecular interactions in 2,6-pyridylacetylenes and their co2(co)4dppm complexes. *J. Organomet. Chem*. **2002**, *648*, 251-269.
3. Pant C S, Wagh R M, Nair J K, et al. Synthesis and characterization of two potential energetic azido esters. *Propellants, Explosives, Pyrotechnics*. **2006**, 31(6), 477-481.
4. Hou Y, Cao S, Li X, et al. One-Step Synthesis of Dual Clickable Nanospheres via Ultrasonic-Assisted Click Polymerization for Biological Applications. *ACS Appl. Mat. Interfaces*. **2014**, 6(19), 16909-16917.
5. Jin F, Zheng M L, Zhang M L, et al. A facile layer-by-layer assembly method for the fabrication of fluorescent polymer/quantum dot nanocomposite thin films. *RSC Adv*. **2014**, 4(63), 33206-33214.
